# Changes in aniseikonia and influencing-factors following successful macula-off retinal detachment surgery

**DOI:** 10.1038/s41598-019-48112-5

**Published:** 2019-08-12

**Authors:** Tomoya Murakami, Fumiki Okamoto, Yoshimi Sugiura, Yoshifumi Okamoto, Takahiro Hiraoka, Tetsuro Oshika

**Affiliations:** Department of Ophthalmology, Faculty of Medicine, University of Tsukuba, Ibaraki, Japan

**Keywords:** Retinal diseases, Medical research

## Abstract

This study investigated the changes in the severity of aniseikonia after surgery for macula-off retinal detachment (RD), and the relationship between aniseikonia and retinal microstructures. The study included 26 eyes of 26 patients undergoing RD surgery. Visual acuity was measured preoperatively, and at 3, 6, and 12 months postoperatively. Degree of aniseikonia and OCT images were obtained at 3, 6, and 12 months postoperatively. The aniseikonia values (mean ± standard deviation) at 3, 6, and 12 months postoperatively were −5.3 ± 4.2%, −4.4 ± 4.4%, and −3.1 ± 3.2%, respectively. Significant improvement was observed from 3 to 12 months postoperatively (*P* = 0.001). Twelve months postoperatively, 14 eyes had micropsia, 1 eye had macropsia, and 11 eyes were free of aniseikonia. Stepwise multiple regression analyses revealed that the severity of aniseikonia at 12 months postoperatively was significantly associated with postoperative development of cystoid macular edema (CME) and epiretinal membrane (ERM), as well as area of preoperative RD. In conclusion, although aniseikonia was gradually relieved after RD surgery during a 1-year follow-up period, approximately half of patients had aniseikonia and almost all of them had micropsia. Aniseikonia was associated with presence of postoperative CME, ERM, and area of preoperative RD.

## Introduction

Advances in vitreoretinal surgical techniques for the repair of rhegmatogenous retinal detachment (RD) surgery during recent years have increased anatomical success rates and improved visual outcomes. However, visual complaints may occur, even after successful and uncomplicated surgery. Aniseikonia is a common postoperative symptom, along with blurred vision and metamorphopsia, observed in patients after RD surgery, with 35% of patients complaining of aniseikonia by questionnaire^1^.

Aniseikonia is a condition of binocular vision in which the size of ocular images between the 2 eyes differs. It can manifest with symptoms including headache, asthenopia, photophobia, reading difficulty, and nausea. Anisometropia due to intraocular lens implantation, penetrating keratoplasty, and refractive error development is a common cause of aniseikonia^2–4^. However, in rare cases, aniseikonia can be caused by retinal diseases^5–10^. A previous report investigated the severity of aniseikonia using the New Aniseikonia Test (NAT) in patients with RD, and found that the absolute value of mean aniseikonia in all patients was 2.3% 6 months after RD surgery, with 45 of 106 patients (42%) having aniseikonia^7^. We followed the time course of aniseikonia changes in patients with epiretinal membranes (ERMs)^8^ and macular holes (MHs)^9^ and found that aniseikonia remained unchanged for up to 6 months after ERM surgery, and improved for up to 12 months after MH surgery. However, to the best of our knowledge, no study has investigated changes in aniseikonia after RD surgery.

Prior studies investigated the relationship between aniseikonia and retinal microstructures using spectral-domain optical coherence tomography (SD-OCT) in patients with retinal disorders^7–9,11^. Inner nuclear layer (INL) thickness was associated with aniseikonia severity in patients with ERM^8^, and MH sizes and external limiting membrane (ELM) defect lengths were associated with aniseikonia severity in patients with MH^9^. In patients with RD, 69% of eyes with aniseikonia exhibited abnormal structures in macular regions (i.e. ERM, hyperreflective ellipsoid zone (EZ), disruption of EZ, cystoid macular edema (CME), MH, and subretinal fluid) after RD surgery^7^, and the difference in central retinal thickness between operated and fellow eyes were associated with aniseikonia severity after pneumatic retinopexy^11^. To date, however, little information is available on the relationship between aniseikonia severity and the foveal microstructure after RD surgery. The purpose of this study was to investigate the changes in the severity of aniseikonia after surgery for macula-off RD, and to evaluate the relationship between aniseikonia and retinal microstructures.

## Results

Fifty-three eyes of 53 patients underwent surgery for macula-off RD. Of these, 33 patients were followed-up for 12 months after surgery. Seven were excluded for the following reasons: 3 had incomplete data, 3 had more than 2.0 diopters of anisometropia postoperatively, and one had ERM before developing retinal detachment. The excluded patient had regularly visited our hospital for ERM before RRD surgery. Macular abnormalities such as ERM were not detected during surgery in the other cases. Thus, 26 eyes of 26 patients were included in the study. Baseline demographic data are presented in Table 1. All patients underwent anatomically successful repair of RD. Among 26 patients, 23 underwent vitrectomy, of whom 13 received combined cataract surgery and vitrectomy, and 3 underwent scleral buckling surgery. No significant intraoperative or postoperative complications were observed, such as retinal re-detachment, choroidal detachment, sub-retinal hemorrhage, or infection. There were no cases of progression of cataract after surgery, and no patients received cataract surgery during the follow-up period. Change in the spherical equivalent from 3 to 12 months was −0.08 ± 0.32 D (mean ± standard deviation). After scleral buckling, it changed to 0.00 ± 0.63 D (mean ± standard deviation, range −0.63D–0.63D). The BCVA of fellow-eyes was −0.03 ± 0.08 (LogMAR, mean ± standard deviation, range −0.08–0.15). There were no relevant findings for the fellow-eyes except mild cataract. Of the 26 patients, 4 were pseudophakic and 22 were phakic in the fellow-eyes. No patients received cataract surgery of the fellow-eyes during the follow-up period.

**Table 1:** Demographic and clinical data of patients with retinal detachment.

Number of eyes	26
Age (years)	58.7 ± 14.8 [19–80]
Gender (men/women)	17/9
Surgical procedures (vitrectomy/scleral buckling)	23/3
Area of retinal detachment (degree)	161 ± 54
Circumferential dimension of retinal tears (degree)	16 ± 13
Duration of symptoms (days)	14.7 ± 23.4 [2–90]
Preoperative BCVA (logMAR)	0.89 ± 0.77
BCVA of fellow-eyes (logMAR)	−0.03 ± 0.08 [−0.08–0.15]

Values are presented as mean ± standard deviation. BCVA = best-corrected visual acuity; logMAR = logarithm of the minimum angle of resolution.

### Changes in visual functions

**Figure 1:**
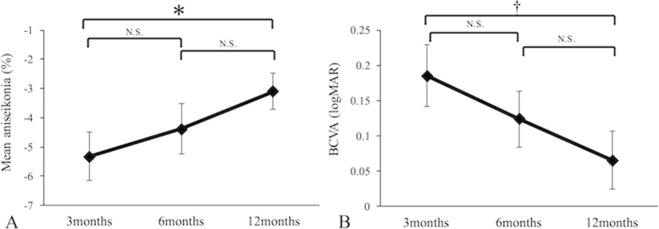
Changes in mean aniseikonia (**A**) and best-corrected visual acuity (**B**) after macula-off retinal detachment surgery. Error bars indicate standard errors. *P = 0.001, ^†^*P* < 0.0001. N.S. = not significant.

**Figure 2:**
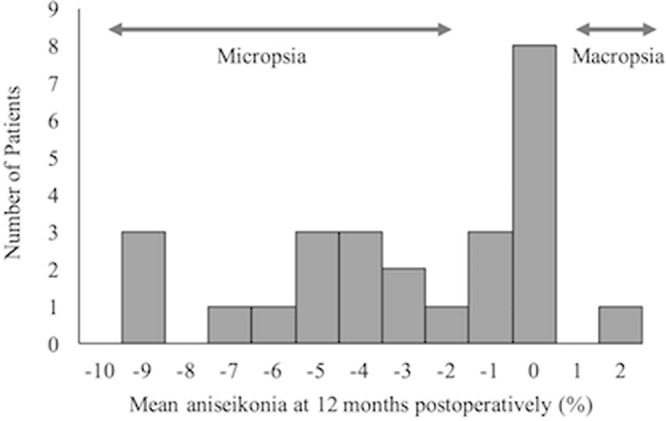
Histogram of mean aniseikonia 12 months after macula-off retinal detachment surgery. Aniseikonia ranged from −9% to +2%. Of 26 patients, 14 had micropsia, and 1 had macropsia.

The mean aniseikonia values (mean ± standard deviation) at 3, 6, and 12 months postoperatively were −5.3 ± 4.2%, −4.4 ± 4.4%, and −3.1 ± 3.2%, respectively. Although mean aniseikonia at 12 months postoperatively significantly improved from 3 months (*P* = 0.001), no significant differences were observed between 3 and 6 months (*P* = 0.129) or 6 and 12 months (*P* = 0.040) (Fig. 1A). BCVA at 3, 6, and 12 months postoperatively were 0.19 ± 0.22, 0.12 ± 0.20, and 0.07 ± 0.21, respectively. Similar results were also found for BCVA (Fig. 1B). At 12 months postoperatively, 14 eyes (54%) had micropsia, 1 eye (4%) had macropsia, and 11 eyes (42%) were free of aniseikonia (Fig. 2).

### Relationship between aniseikonia and BCVA

Three (*r* = −0.336, *P* = 0.093), 6 (*r* = −0.234, *P* = 0.241), and 12 (*r* = −0.137, *P* = 0.493) months postoperatively, mean aniseikonia showed no correlation with BCVA. Mean aniseikonia at 12 months postoperatively was unassociated with preoperative BCVA (*r* = −0.193, *P* = 0.334).

### Relationship between visual functions and influential factors

**Figure 3:**
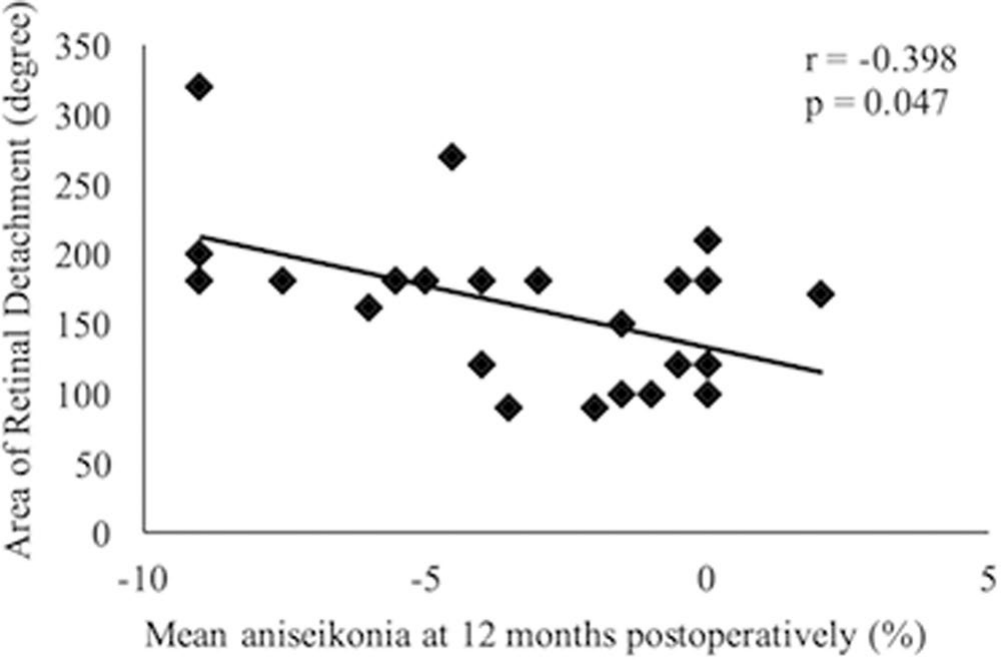
Correlation between mean aniseikonia 12 months after macula-off retinal detachment surgery and area of retinal detachment.

Table 2 lists the correlations between visual functions 12 months postoperatively, with OCT parameters and RD characteristics. Mean aniseikonia 12 months postoperatively was significantly associated with postoperative development of CME after surgery, as well as preoperative RD area (Fig. 3); other variables were not relevant. The range of duration of symptoms was 2–90 days. The longest case (90days) had severe aniseikonia after 12 months surgery (−9%), however duration of symptoms was not associated with aniseinkonia (Table 2). BCVA 12 months postoperatively was significantly associated with postoperative development of CME, and disruption of ELM and EZ 12 months postoperatively. Stepwise multiple regression analyses revealed that mean aniseikonia 12 months postoperatively was significantly associated with postoperative development of CME (*r* = −4.67, *F* = 21.99), preoperative RD area (*r* = −0.033, *F* = 20.00), and postoperative development of ERM (*r* = 2.38, *F* = 7.12). BCVA 12 months postoperatively showed a significant association with ELM status (*r* = −0.459, *F* = 13.86).

**Table 2:** Correlations between visual functions at 12 months and various parameters.

Factors		Number	Mean aniseikonia	p value	BCVA (logMAR)	p value
ELM disruption	(+)	2	−3.0 ± 3.5	0.966	0.49 ± 0.47	0.001^*^
	(−)	24	−3.1 ± 3.3		0.03 ± 0.14	
EZ disruption	(+)	4	−5.0 ± 3.5	0.205	0.33 ± 0.35	0.003^*^
	(−)	22	−2.8 ± 3.1		0.02 ± 0.13	
IZ disruption	(+)	12	−3.5 ± 2.8	0.606	0.12 ± 0.26	0.211
	(−)	14	−2.8 ± 3.6		0.02 ± 0.13	
Presence of CME	(+)	5	−6.6 ± 2.4	0.004^*^	0.24 ± 0.34	0.036^*^
	(−)	21	−2.3 ± 2.8		0.03 ± 0.15	
Presence of SRD	(+)	4	−1.4 ± 1.9	0.253	−0.01 ± 0.14	0.465
	(−)	22	−3.4 ± 3.3		0.08 ± 0.22	
Presence of ERM	(+)	7	−2.1 ± 3.1	0.370	0.18 ± 0.30	0.084
	(−)	19	−3.4 ± 3.3		0.02 ± 0.15	
**Factors**			**Mean aniseikonia**		**BCVA** (**logMAR**)	
			**r**	**p value**	**r**	**p value**
Area of retinal detachment			−0.398	0.047^*^	0.080	0.690
Circumferential dimension of retinal tears			−0.055	0.783	0.017	0.931
Duration of symptoms			−0.145	0.468	−0.028	0.891

^*^Significant at p < 0.05.

Values are presented as mean ± standard deviation; BCVA = best-corrected visual acuity; logMAR = logarithm of the minimum angle of resolution; ELM = external limiting membrane; EZ = ellipsoid zone; IZ = interdigitation zone; CME = cystoid macular edema; SRD = serous retinal detachment; ERM = epiretinal membrane; r = regression coefficient.

## Discussion

Nearly half of patients had aniseikonia 12 months after RD surgery, and mean aniseikonia 12 months postoperatively was −3.1%, ranging from −9% to + 2%. Clinically, symptoms were induced when the percentage of aniseikonia was greater than 3–5%; it is generally accepted that 5% aniseikonia is the limit of tolerance to permit fine stereoptosis^12,13^. In this study, 13 of 26 patients (50%) showed aniseikonia of −3% or less and 8 of 26 patients (31%) showed aniseikonia of −5% or less. Thus, it is probable that quality of vision remains deteriorated in some patients due to severe aniseikonia, even one year after surgery.

Mean aniseikonia continued to improve for up to 12 months after macula-off RD surgery (−5.3 ± 4.2%, −4.4 ± 4.4%, and −3.1 ± 3.2%, at 3, 6, and 12 months, respectively). Previous studies assessed aniseikonia after surgery for RD^7,14–16^, ERM^5,6,8^ and MH^9^. We investigated changes in aniseikonia after surgery for ERM^8^ and MH^9^, and found that absolute mean aniseikonia did not change after surgery for ERM (preoperative and 3 and 6 months postoperative values were 6.2%, 6.2% and 6.5%, respectively) and continued to improve for up to 12 months after surgery for MH (preoperative and 3, 6, and 12 month postoperative values were 3.8%, 1.2%, 1.5%, and 1.0%, respectively). In terms of aniseikonia, patients with macula-off RD have a better prognosis than patients with ERM, but a worse prognosis than patients with MH. The discrepancy between macula-off RD and ERM seems to be associated with disease duration. In patients with ERM, the period from onset to surgery tends to be longer than in patients with RD, because visual function is not impaired at an early stage in ERM. Thus, photoreceptors are compressed for a long time, resulting in no improvement to aniseikonia. The discrepancy between RD and MH seems to be associated with difficulties in restoring the photoreceptor distribution. MH develops when retinal photoreceptors move centrifugally, thus the distribution of the photoreceptors is more likely to be restored by surgery. On the other hand, in RD patients, a previous study reported that macula displacement is common after surgery for RD^17^, thus restoring photoreceptor distribution by surgery is challenging.

We found that 15 of 26 patients had aniseikonia 12 months postoperatively, and almost all of them (14 of 15 patients) had micropsia, consistent with previous reports^7,11^. Okamoto *et al*. reported that approximately half of patients with macula-off RD developed micropsia, whilst only 10% presented with macropsia^7^. Lee *et al*. reported that 88% of patients had aniseikonia after pneumatic retinopexy, and all of them had micropsia^11^. It is thought that retinal induced aniseikonia may occur after stretching or compression of the retina^5^. If photoreceptors are stretched, the image would stimulate fewer cells and the object perceived as smaller (micropsia). On the other hand, if photoreceptors are compressed, the image would stimulate more cells and the object would be perceived as larger (macropsia). It is known that macular edema^10,18^, MH^9^, and central serous chorioretinopathy^19^, in which the retina is stretched, accompany micropsia. In this study, postoperative aniseikonia was significantly associated with postoperative development of CME. Sjöstrand *et al*. investigated aniseikonia after surgery for macula-off RD and found that micropsia was present in 3 of 7 patients and transient CME could be detected in 2 of these cases^15^. We previously investigated aniseikonia and microstructure after RD surgery, and reported that 21% of eyes with micropsia exhibit CME^7^. Stretching photoreceptors due to CME may play a key role in the development of aniseikonia after surgery for macula-off RD.

Postoperative aniseikonia was also associated with RD area. Previous reports investigated unintentional displacement of the retina after vitrectomy for rhegmatogenous RD, using fundus autofluorescence imaging. They reported that RD area was significantly associated with postoperative retinal displacement^17,20^. We speculate that such displacement of the retina may stretch photoreceptors, resulting in micropsia.

Stepwise multiple regression analyses revealed that mean aniseikonia was associated with the presence of ERM, CME, and RD area. In this study, patients with ERM after macula-off RD surgery had milder micropsia than those without ERM. Previous studies reported that most of patients with ERM had macropsia^5,6,8^. Thus, ERM-induced macropsia may mask micropsia after surgery for macula-off RD.

It has previously been reported that scleral encircling significantly correlates with changes in myopia^21^. In this study; however, the three eyes underwent circumferential scleral buckling instead of scleral encircling, and the change in the spherical equivalent after the procedure was relatively small. We concluded that the change in refractive error has little influence on aniseikonia.

Limitations to the current study include the small sample size, few observation points, and short follow-up period. It was difficult to determine whether presence of macular abnormalities was the result of surgery or existed prior to it because OCT images were not recorded before surgery. The first postoperative observation point was 3 months after surgery. Thus, macular abnormalities (i.e., CME or abnormality of retinal outer line) which resolve in the early postoperative periods may be overlooked. Transient macular abnormalities may have influenced the results. Sjöstrand *et al*. investigated aniseikonia after surgery for macula-off RD, reporting that 2 of 7 patients still had micropsia approximately 3 years after surgery^15^. Judging from previous reports and our present study, long-standing aniseikonia could occur even after successful surgery for macula-off RD. Thus, further studies with larger sample sizes, more observation points, and longer follow-up periods are needed.

In conclusion, although aniseikonia was gradually relieved after RD surgery during a 1-year follow-up period, approximately half of patients had aniseikonia and almost all of them had micropsia. Aniseikonia was associated with presence of postoperative CME, ERM, and area of preoperative RD.

## Methods

We studied a series of consecutive patients who underwent surgery for primary unilateral macula-off RD at the University of Tsukuba Hospital, between July 2011 and September 2013. Patients were followed-up for 12 months after surgery. This prospective study was approved by the Institutional Review Board of the Tsukuba University Hospital, and was conducted in accordance with the tenets of the Declaration of Helsinki. Signed informed consent was obtained from all study subjects after the nature of the study had been explained to them. Patients were excluded if they had: undergone previous vitreoretinal surgery, any ophthalmic disorders (except mild cataract or refractive errors), anisometropia of more than 2.0 diopters postoperatively, RD complicated by proliferative vitreoretinopathy or resulting from giant retinal tears, macular hole, or ocular trauma.

We examined visual acuity before and at 3, 6, and 12 months postoperatively, and evaluated the degree of aniseikonia and retinal microstructure at 3, 6, and 12 months postoperatively. Best-corrected visual acuity (BCVA) was measured with the Landolt Chart, and expressed as a logarithm of minimal angle resolution (logMAR). Degree of aniseikonia was quantified using the New Aniseikonia Test (NAT; Handaya, Tokyo, Japan). The NAT consists of plates containing matched pairs of semicircles (one red and one green). The diameters of the semicircles vary in 1% of the steps; semicircles of different sizes are arranged in pairs along a series. The subjects viewed the plates from a distance of 66 cm with appropriate correction. The subject wears red/green spectacles and views the plates to allow the right eye to see one semicircle and the left eye to see the other semicircle, in each pair. The subject identifies the pairs in which both semicircles appear of equal size. The size difference between the semicircles in each pair represents the percentage of aniseikonia. Measurements were repeated three times. We quantified the degree of aniseikonia in horizontal and vertical meridians, and the mean values were used for data analysis. We defined macropsia as a mean aniseikonia of +2% or more, and micropsia as a mean aniseikonia of −2% or less. Patients with logMAR BCVAs of >1.0 were excluded because it was difficult to perceive the semicircles^22^.

SD-OCT (Cirrus high-definition OCT; Carl Zeiss, Dublin, CA) images were recorded after pupil dilation. Horizontal 5-line raster scan protocols were performed for each eye with Cirrus analysis software version 3.0. Scans with signal strengths higher than 6/10 were considered appropriate. OCT images were used to assess ELM, EZ, and interdigitation zone (IZ) status at 12 months postoperatively, and presence of CME, serous retinal detachment (SRD), and ERM were evaluated at 3, 6, and 12 months postoperatively. Diagnose of line disruption was made based on loss of hyperreflective line, within 1-mm area, centered at the presumed fovea. Two graders (YS, TH), masked to the patients’ clinical findings, including visual acuity and degree of aniseikonia, assessed the status of ELM, EZ and IZ line, and presence of CME, SRD and ERM.

Clinical data were collected, including age, sex, surgical procedures (vitrectomy or scleral buckling), area of retinal detachment, circumferential dimension of retinal tears, and duration of symptoms. The area of RD was measured during surgery.

Surgeries were performed at our hospital by 3 vitreoretinal surgeons (FO, YO, YS). Standard three-port 25-gauge pars plana vitrectomies were performed; the surgical technique comprised a vitrectomy that released vitreous traction around the breaks, internal drainage of the sub-retinal fluid, total gas-fluid exchange with air or 20% sulfur hexafluoride (SF_6_), and end-laser photocoagulation. In eyes with clinically significant cataracts, the lens was removed by phacoemulsification and intraocular lenses were implanted, followed by vitrectomy. In no case was the internal limiting membrane removed. When scleral buckling was performed, cryopexy and circumferential silicone sponge buckling were used to support the retinal breaks. Air injections were performed when required. In both group, all patients injected with gas were instructed to maintain a face-down position for 3–7 days.

Mean scores and standard deviations for each parameter of visual functions were calculated. One-way repeated-measures analyses of variance (ANOVA) with Bonferroni/Dunn post-hoc tests were used to assess changes in visual functions (aniseikonia and BCVA) after surgery. The relationships between degree of aniseikonia and BCVA were examined using Spearman’s rank correlation tests. Unpaired t-tests were used to compare postoperative visual functions between the two groups, based on the status of ELM, EZ, and IZ at 12 months postoperatively, and the presence of CME, SRD and ERM after surgery. We used Spearman’s rank correlation tests to examine the relationships between visual functions and circumferential dimensions of retinal tears, RD areas, and symptom durations. Multivariate analyses with stepwise regressions were used to determine parameters significantly relevant to mean aniseikonia 12 months postoperatively. All tests of association were considered significant if *P* < 0.05, except for the Bonferroni/Dunn post-hoc tests, which were considered significant if *P* < 0.0167. All analyses were conducted using StatView (version 5.0, SAS Inc., Cary, NC, USA).
